# Conscious engagement within patients’ and simulated participants’ personal space: medical students' perspective

**DOI:** 10.1186/s41077-022-00224-1

**Published:** 2022-09-05

**Authors:** Chara Banks-McGovern, Gerard J. Gormley, Diane Wilson, Grainne P. Kearney

**Affiliations:** 1 Centre for Medical Education, Queen’s University Belfast, Whitla Medical Building, 97 Lisburn Road, Belfast, BT9 7BL Northern Ireland; 2 Clinical Skills Education Centre, Queen’s University Belfast, Belfast, Northern Ireland

**Keywords:** Medical education, Phenomenology, Personal space, Clinical gaze

## Abstract

**Background:**

*#MeToo* prompted a shift in acceptable societal norms, sparking global recognition of the complexities of entering another’s personal space. Physical examinations are an integral part of medicine yet have the capacity to encroach upon patient’s personal space, whether in simulated or clinical environments. Examinations may be misconstrued as inappropriate advances, with negative effects for both patient and doctor. Medical educators must consider how they teach students to approach this complex task. This study aimed to gain insight into the lived experiences of medical students when working within patient’s personal space. This builds on previous research from the perspective of simulated participants.

**Method:**

A hermeneutic phenomenology approach was used to explore lived experiences of working within patient’s personal space. Data was collected from seven medical students through semi-structured interviews and thematically analysed using template analysis.

**Results:**

The analysis yielded four main themes: (1) transitioning into a privileged position; (2) negative role modelling: emphasising the physical; (3) consent: a dynamic and fragile state; and (4) a simple act or a complex performance?

**Discussion:**

This study provides a unique insight into the lived experiences of medical students when working within a patient’s personal space. The physical examination is a complex process; the experiences of medical students can shape learning on crossing boundaries. Medical educators need to reflect this complexity in teaching, mirroring societal interest around the boundaries of consent. Students need a pedagogical space to develop these interpersonal skills, to prevent early adoption of the *clinical gaze*, and to create more consciously engaged doctors for the future.

## Background

The *#MeToo movement is* an avenue for people to share their trauma of sexual harassment and assault. The movement revived in 2017, when further light was shed on the scale of sexual harassment hidden in plain sight in society. This prompted a shift in acceptable societal norms and sparked global recognition of the complexities of consent and personal boundaries [1]. Society is intolerant of the unsolicited breaking of personal boundaries; the education of medical professionals needs to reflect this same intolerance.

Respecting a patient’s perspective of their personal space is integral in clinical care. An absence of clear communication involving the boundaries of this space can lead to physical examinations being misconstrued as inappropriate advances. There have been a number of high-profile cases of such failings in practice. For example, in 2011, a doctor was accused of inappropriate advances when auscultating a female patient’s chest. After a prolonged investigation, the doctor was vindicated. Yet, the consequences of a permanent breakdown of trust between patient and doctor remain [2]. It is clear that medical education must evolve apace with a more mindful society following the *#MeToo* movement in order to reduce these occurrences.

At the beginning of their educational journey, medical students need experience of and teaching on the crucial expertise behind a safe and mutually respectful physical examination. Simulated learning environments can help here. Simulated participants (SPs) are increasingly involved in such teaching whereby they can simulate a patient’s illness and present their illness in a standardised way [3, 4]. In a controlled simulated learning environment, students are afforded a mutually comfortable space to learn clinical skills in confidence. Simulated learning can offer a space for students to consider how to agree consent for the first time and consider what is involved in learning intimate examinations [5–8]. This safe space creates a standardised and more replicable learning environment. But does the benefit of reproducibility outweigh any possible risk of creating doctors who are less consciously engaged with the humanistic side of the interaction outside of this manufactured environment?

Working with simulated participants offers unique benefits which can be difficult to obtain in “real-world” clinical scenarios. Insight can be obtained from this professional group into boundary-crossing when interacting with medical students. One study explored the negotiation of boundaries involved in the physical examination from the point of view of the SPs. To facilitate the learning experience for students, the SPs accepted the discourse of the clinical gaze allowing the student to breach the agreed boundaries and be positioned subordinately to the student [9]. The *clinical gaze* is when the clinical aspects of care are separated from the patient as a whole [10]. It was evident from this study that further research was required from the perspective of medical students. If we understand the perspective of medical students in this same moment, perhaps we could prevent further adoption of this dehumanising clinical gaze.

This research aimed to develop an understanding of the fragile nature of working within a patient’s personal boundaries during the physical examination from the viewpoint of medical students. The perspective from this generation of future doctors could be used to inform teaching to minimise any breakdown of trust or misunderstandings of consent. As a more conscious society, we need to tailor teaching to embody this. Medical education must emulate the ever-evolving understanding of what constitutes a patient’s personal space. Insights from this study can govern the promotion of a safe learning environment for both patient and student, optimising the comfort of both parties, before students bring their learning into clinical environments.

## Methods

Ethical approval was obtained from the Faculty of Medicines Health and Life Sciences Research Ethics Committee (MHLS 20_158).

### Conceptual orientation

To explore medical students’ lived experiences in this area, hermeneutic phenomenology was chosen. This lens provides a means to gain a nuanced insight into the phenomena of the experience, rather than focus on an external reality that can be described objectively and physically. Phenomenology can be divided into descriptive and interpretive. Interpretive, such as hermeneutic phenomenology, involves the interpretation of the meaning of a phenomenon to understand it. This involves engaging with the data interpretively to search for themes whilst refraining from formalising the analytical method. This means the phenomena can lead the direction in how the data is analysed [11].

### Setting

The study was based in the Centre for Medical Education at Queen’s University Belfast (QUB). All interviews took place over Microsoft Teams due to COVID-19 restrictions. Students undertaking their undergraduate medical degree at QUB follow an integrated curricular model spanning 5 years. Teaching in the first 2 years is largely university-based lectures with some clinical contact. The latter years of teaching are clinically based. The research team consisted of CMcG (a medical student undertaking their intercalated year) and GK, GG, and DW who are clinical academic general practitioners. GG is also a professor of simulation.

### Data collection

All third- and fourth-year medical students were invited to participate in the study by email (*n* = 544). QUB students experience an increase in clinical contact from the third year onwards, therefore offering a greater variance in experience of working within patients’ personal space. A convenience sampling strategy was used for recruitment. As typical with phenomenological research, there were a small number of participants recruited with the initial aim of recruiting eight participants, to gain a deeper insight into the experience of the medical students whilst ensuring a broad scope of experiences, without an overwhelming amount of data to prevent detailed analysis. Seven participants took part, four male and three female. Participant’s age ranged from 21 to 26 with an average age of 23. All participants were interviewed on a one-to-one basis in semi-structured interviews by CMcG, allowing the interviews to be exploratory of the student’s experience. Students were asked to share their experience of examining patients and how they felt approaching the patient’s personal space. They were also asked about their perception of what can make examining a patient feel uncomfortable and how this can be improved. The question guide was informed from prior evidence from the research teams’ knowledge and a literature review undertaken by CMcG. This guide was used to open dialogue in the interviews as participants lead the direction with explorations of their own experience, in keeping with the philosophical approach. Participants provided examples from their collective experience working within patients’ personal space varying from university-based examples involving simulated participants to “real patients” in primary and secondary care. After the interview, each recording was transcribed verbatim, anonymised using pseudonyms, and checked for accuracy.

### Analysis

The transcriptions from the interviews were the focus of the analysis. Template analysis was used to complement the hermeneutic phenomenological approach. The researchers were continually aware of the research question and remained focused on this throughout. Before undertaking the analysis, the research team completed a reflexivity log to bring forward any assumptions around the topic which could influence the interpretive process. Conscious acknowledgment of the research team’s own experience through dialogic and written reflection occurred continually. Before the analysis, a literature review was undertaken by CMcG. Tentative, a priori themes were created from within the research teams understanding of the literature. In response to continued analysis of further transcripts, these codes were modified throughout. Codes that reoccurred were grouped to design an initial research template. This template then was continually modified once the remaining transcripts were analysed. Ultimately, all transcripts were coded against the conclusive template to embody the hermeneutic circle. The conclusive template encompassed the entire data, establishing experience by discovering and interpreting phenomena [11]. The research team all approved the final themes.

## Results

Over 200 min of interview data was collected. Analysis of data constructed four main themes: (1) transitioning into a privileged position, (2) negative role modelling: emphasising the physical, (3) consent: a dynamic and fragile process, and (4) a simple act or a complex performance? Participants refer to “patients” as a collective for SP and real patients unless stated otherwise.

### Transitioning into a privileged position

Participants reported experiencing a transition in their societal status when embarking on their educational journey to become a doctor. Entering a patient’s personal space provided a particular instance of this transition which whilst highly formative was simultaneously fraught with risk. Entering any individuals’ personal space after discussion and agreement should be a respectful, mutually beneficial experience. However, this act equally has the potential for negative connotations as egregious as assault. Participants reported recognising that an instant in an examination could have the potential to change a lifetime for both the patient and the student. As they transition professionally, medical students experience an evolution of their own perceived right to enter an individual’s personal space, starting from a humble and even uncomfortable baseline.

*“You kind of have to remind yourself that you’re not doing anything ****illegal****, you are supposed to be there.”****Mary***

Participants transition to what they consider to be the privileged state of having the opportunity to examine a patient for their direct learning. This educational, privileged state can only be facilitated by patients offering their personal space for examination. Participants acknowledged how readily patients extended the boundaries of their personal space to create a learning opportunity for them.

*“you’re not another member of staff coming to look after them, they’re helping you”****Hannah***

*“They’re always thinking that you are the next generation of doctors. You have so many patients who just seem excited that they can be a part of your learning.”****Paul***

This transition from inexperienced to privileged helped students feel some legitimacy in learning important and complex negotiations.

### Negative role modelling: emphasising the physical

Participants disclosed placing more emphasis on the physical dimensions of examinations with little reference to the interpersonal aspects. They described prioritisation in applying the physical aspects of an examination with their feedback largely reflecting this. Participants continually referred to these physical aspects as “the basics” often describing the act of examining a patient mechanically. This involved a rigid process in which steps were followed methodically, as a tick box exercise exposing early acceptance of the clinical gaze.

*“I’m quite methodical, I usually have a list of what I want to do in my head and then I just check it off as we go.”****Anna***

Participants shared experiencing a drive to improve their skills to achieve success in their exams and complete their logbooks. They reported no such drive to improve their interpersonal skills as these are more subjective and difficult to assess.

Participants referenced the contrast in their teaching from the university setting to what is experienced on placement. They shared a view that senior doctors often role-modelled less than optimal humanistic skills when interacting with patients. Participants also reported experiencing the view that doctors act out the clinical gaze with time, describing their perception that this is just part of the job.

*“If a doctor has been in the job for so long and they sort of have lost that instinct or a bit of empathy where they think I just have to get on with it, this is my job.”****Will***

This problematic perception of acceptance of the clinical gaze, as part of the job, can transcend into the next generation of doctors, through the dangers of poor role modelling.

### Consent: a dynamic and fragile process

Participants expressed experiencing a dynamic interaction whilst working within a patient’s personal space. A dialogic process occurred when gaining consent from a patient, in which students and patients continually collaborate in mapping a mental framework for the examination. This framework was based on a shared imagined concept of what was expected and desired by both parties, further detailing the nature and context of the examination. This is the basis of the negotiation of power that exists in informed consent which participants reported as integral to maintaining a patient’s trust.

*“Informed consent, making sure the patient ****actually ****understands what your aim is.”****Mary***

Participants shared that an individual’s construct of what constitutes their personal space varies with every examination. Participants described the possibility of differing assumptions around this space, which could be problematic if not recognised. They reported experiencing that any examination can encroach upon a patient’s personal space.

*“Any physical exam in my opinion is breaking a patient’s personal space one way or the other, no matter how invasive or non-invasive it is.”****John***

Although participants showed an understanding of consent, they often referred it to as a simple step in examinations. Gaining consent was often quickly listed with asking a patient’s name and date of birth as the first steps in examining a patient.

*“First, cleanliness, checking name, date of birth and consent.”****Mary***

Participants also described negotiations of consent in more intimate examinations and the importance of obtaining a chaperone as an extra “step” to follow in these exams.

*“there are extra steps that you need to have in your head beforehand”.****Nathan***

Participants reported experiencing a change in the power dynamic when a senior doctor gained consent on their behalf. They believed these situations influenced the balance of power and partnership that would normally be experienced if it were the students gaining consent as described above. Whilst attempting to step into a patient’s lifeworld, participants expressed the belief that patients may feel more challenged in confronting a doctor than a student. Participants reported in their experience that patients behaved in a more subservient manner to senior staff.

*“They feel like they have to say yes because these people are looking after them.. they feel sort of indebted to them in a way.”****Paul***

### A simple act or a complex performance?

Participants shared experiencing the act of examining a patient to be a complex, multifactorial process, reliant on a heterogenous range of influences. This is complicated further by being an alien experience for students. Participants reported that they would commonly rehearse these examinations in their minds, focusing on the physical dimensions as described above. Participants shared the belief that this cognitive overload left little attention available to the interpersonal side of the interaction.

*“… trying to remember everything, while also trying to be mindful of the patient while the doctor is critiquing you step by step.”****Paul***

Students experience a complex orchestration of both intra- and interpersonal dialogue when interacting with a patient. They reported experiencing a sense of instilled trust when viewed as part of the medical profession, having transitioned into this privileged position. This assumed trust was experienced in conflict with an internal narration of stress and insecurity. Participants shared their experience of self-awareness of their emotional state whilst simultaneously trying to hide this from their patients. They reported a felt need to portray a state of confidence as a form of impression management. This need to perform and portray a calm demeanour is for the benefit of the patient even when conflicting with their internal state. Participants believed that without this performance, patients would assume the student lacked competency, resulting in an uncomfortable interaction for the patient.

*“If you look like you don’t know what you’re doing. Then that doesn’t instil confidence in the patient.”****Nathan***

This complex performance of falsified confidence adds further to the cognitive overload faced by students in these novel experiences.

## Discussion

### Learning to tick boxes

This study highlighted that the lived experience of performing a physical examination by medical students is a complex, dynamic interaction, heightened further by the intricacies of working in a patient’s personal space, even in a SP encounter. Examinations require clinicians to be consciously engaged with the complexities of the humanistic interaction. During their transition to becoming a doctor, students are faced with mastering the physical concept of an examination. This interaction is often taught as a transferable skill in manufactured and created learning environments such as simulation. Yet, this controlled teaching style could prompt mechanical behaviour where students follow a mental checklist whilst examining a patient, attending to what they consider to be *the basics.* This checklist approach is further reflected in teaching guidelines for examinations, which are often devised step by step. Students have reported finding these interactions in controlled teaching styles to be unnatural, leading to a sense of rigidity [12]. This rigid, mental checklist can be dangerously carried into examinations in the working world creating less consciously engaged doctors disregarding the humanistic side of patient interaction.

Students recognised that it was their prerogative to improve their humanistic skills as part of the “hidden curriculum” [13]. They were motivated to fill their logbooks and pass their assessments reflecting the well-used “truism” in medical education that “assessment drives learning”. Students who are required to fill in logbooks as a motivating factor have a 30% increased exposure to practical procedures [14]. No equivalent driving force has been designed for students to improve their humanistic skills, even though these skills are recognised as integral to improving patient care [15]. More explicit teaching on these skills is needed particularly for the less consciously engaged students who may struggle with awareness of humanistic skills, when working within patients’ personal space. To awaken this desire to develop these crucial humanistic skills, medical education needs to recognise these skills as the fundamental *basics* and create formative experiences to learn in and reinforce in clinical practice.

### Consequences of cognitive overload

Students expressed a sense of occupying a liminal place in society. They are developing their professional identity as part of the medical collective [16]. Whilst forming this professional identity, students reported assuming a position of privilege in which they are permitted to examine a patient to further their education. Societal influences of gratitude to the medical profession can influence this interaction between patients and doctors in training. Even trained SPs can subordinate to students, losing their agency and offering their personal space to facilitate learning [9, 17]. Students must balance these competing roles of being an aspiring yet humble learner, reflecting the wider societal need not to abuse positions of power as learnt from the *#MeToo movement*.

Students reported feeling a need to fulfil their incipient professional identity as a doctor, through portraying confidence. We found students felt a great sense of discomfort when working within patients’ personal space arising from unfamiliarity in the clinical environment. When students experience a lack of confidence, they more readily accept the discourse of the clinical gaze and adopt checklist behaviour. These stress-inducing situations result in cognitive overload for students, leaving little attention for the complexities of the interpersonal reaction.

### Pedagogical implications

The shaping of the ability of medical students to safely and considerately work within a patient’s personal space is greatly influenced by the method of teaching used. University-based teaching through controlled environments can reinforce an unbalanced emphasis on the physical dimensions of examinations, with students adopting the clinical gaze. This is reinforced by previous research showing SPs accepting the discourse of the clinical gaze and becoming subordinate to students in these moments [9]. To create more consciously engaged doctors reflecting today’s society, the limitations of this mechanical checklist behaviour should be openly discussed with students. Educators may benefit from discussing their own adoption of the clinical gaze and how the dissemination of this in teaching could be prevented. When teaching medical students in simulation or with the help of SPs, an emphasis should be placed on both the physical and humanistic side of the interactions lending to more consciously engaged practitioners in the future.

Current teaching works on the assumption that students will have clinical experiences in the future to further develop the interpersonal skills required for working within a patient’s personal space. Students need a pedagogical or simulated space to develop their professional identity, which embeds a focus on both physical and humanistic skills. The current practice appears to place a priority on learning the physical aspect of examinations. If teaching placed greater emphasis on intertwining physical and humanistic skills, we could help students to extend what they consider to be *the basic*s. In turn, this could help rebalance the cognitive load through confidence with practice, discouraging the adoption of the clinical gaze. The teaching of these skills should no longer be part of the hidden curriculum, dependent solely on a student’s awareness of and desire for conscious engagement.

### Strengths and limitations

Whilst aiming for transferability, this study, like all qualitative research, has its limitations. This study was UK based and therefore it may not be representative of some other medical education settings. Members of the research team included clinical teachers who may be known to some participants. However, all interviews and correspondence were carried out by an intercalating medical student, allowing participants to speak freely about teaching practices. Analysis was an iterative process, in which reflexivity logs were established to ensure that researchers were aware of potential bias throughout. The inclusion of a medical student on the research team strengthened the data collection and analysis process, by offering a variance in the experience of the research topic.

More research into the experience of medical students when working within a patient’s personal space is needed to continue to guide teaching in this important area. This research highlighted the entanglement of intra- and interpersonal dialogue faced by students when working within a patient’s personal space. This generation of students, influenced by societal discourses such as the *MeToo* movement, will rightly demand more in-depth exploration in this area. Future theoretical insights may include the perspectives of more senior students, those who recently have graduated, those of the experienced doctors, and doctor/patient gender differences during such close physical examinations.

## Conclusion

Today’s society is consciously engaged with considering the boundaries of consent. Medical teaching needs to reflect this, creating doctors who are attentive to individual patient’s personal space and their role in permitting interactions. Current teaching methods appear to place a greater significance on the physical aspects of an examination. Students are faced with undertaking what may appear to be a simple task in teaching, often simulated environments; the reality in clinical practice is complex and influenced by a heterogenous range of factors. For students, this novel juggling act can result in cognitive overload, in which learnt priority of the physical aspects upstages the delicate interpersonal skills needed. Mechanistic behaviour can result from this, with students becoming checklist focused and adopting the clinical gaze when working within patients’ personal space. Students need to be given a pedagogical space to consider these interpersonal skills as *basics* and become consciously engaged doctors in the future.

## Data Availability

The datasets used and analysed during the current study are available from the corresponding author on reasonable request.

## Abbreviation

SP: Simulated participant
